# Cellular Signaling Pathways Activated by Functional Graphene Nanomaterials

**DOI:** 10.3390/ijms19113365

**Published:** 2018-10-27

**Authors:** Anna Piperno, Angela Scala, Antonino Mazzaglia, Giulia Neri, Rosamaria Pennisi, Maria Teresa Sciortino, Giovanni Grassi

**Affiliations:** 1 Department of Chemical, Biological, Pharmaceutical and Environmental Sciences, University of Messina, V.le F. Stagno d’Alcontres 31, 98166 Messina, Italy; ascala@unime.it (A.S.); nerig@unime.it (G.N.); rpennisi@unime.it (R.P.); mtsciortino@unime.it (M.T.S.); ggrassi@unime.it (G.G.); 2 CNR-ISMN c/o Department of Chemical, Biological, Pharmaceutical and Environmental Sciences of the University of Messina, V.le F. Stagno d’Alcontres 31, 98166 Messina, Italy; amazzaglia@unime.it; 3 Department of Chemistry, Materials, and Chemical Engineering “Giulio Natta”, Politecnico di Milano, 20131 Milan, Italy; giulia.neri@polimi.it

**Keywords:** graphene/cell interactions, graphene uptake, graphene signaling, nanomaterials, doxorubicin/graphene

## Abstract

The paper reviews the network of cellular signaling pathways activated by Functional Graphene Nanomaterials (FGN) designed as a platform for multi-targeted therapy or scaffold in tissue engineering. Cells communicate with each other through a molecular device called signalosome. It is a transient co-cluster of signal transducers and transmembrane receptors activated following the binding of transmembrane receptors to extracellular signals. Signalosomes are thus efficient and sensitive signal-responding devices that amplify incoming signals and convert them into robust responses that can be relayed from the plasma membrane to the nucleus or other target sites within the cell. The review describes the state-of-the-art biomedical applications of FGN focusing the attention on the cell/FGN interactions and signalosome activation.

## 1. Introduction

**Figure 1:**
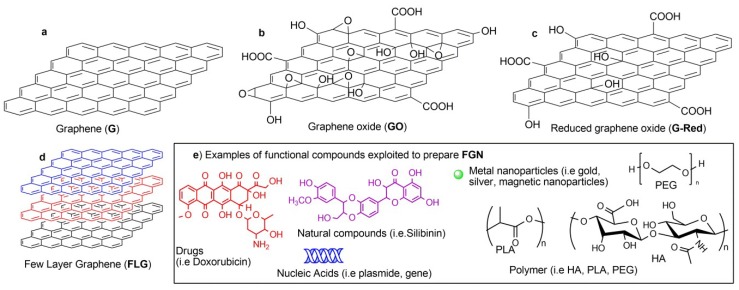
The summary of structural models of various graphene derivatives. (**a**) Pristine graphene (G), (**b**) graphene oxide (GO); (**c**) reduced graphene oxide (G-Red); (**d**) few-layer graphene (FLG); (**e**) some examples of functional compounds exploited to prepare Functional Graphene Nanomaterials (FGN).

Graphene (G) is an sp^2^ hybridized model of carbon atoms in a repeated manner, forming a regular lattice structure and their electrons participate in aromatic conjugated domains (Figure 1). Since its discovery in 2004, a rapidly growing number of graphene-related materials, including few-layer graphene (FLG), graphene oxide (GO), reduced graphene oxide (G-Red), and functionalized graphene (f-G) have been developed [1] and proposed for applications ranging from electronics, energy, bioremediation, nanomedicine [2,3,4]. Among the different subtypes of G-based nanomaterials, GO has been more investigated compared to other G materials (e.g., pristine G and reduced G). GO is highly oxidized with a large number of residual epoxide, hydroxyl, and carboxylic acid groups on its surface, which have been widely exploited to obtain a variety of functionalized graphene-based materials. The presence of a large concentration of ionizable oxygen functionalities as well as the chemical nature of these functionalities make GO dispersible, according to the specific pH-values, and biocompatible. However, the damage of sp^2^ network impairs the electronic and optical properties, limiting some kind of applications (i.e., imaging).

The reduction of GO to G-Red restores the structure and properties of G. In comparison with GO, G-Red is known to have an increased conductivity, and mechanical strength, the fact that makes it a more attractive tool for applications in theranostic nanomedicine. However, the π–π stacking of aromatic rings and van der Waals interactions limit its dispersion in a biological medium and induce aggregation, so only functionalized pristine G or functionalized G-Red can be used in nanomedicine. The chemical functionalization of graphene-based materials (i.e., G, GO, or G-Red) tunes the structural, physical, chemical and electronic properties together with the biological responses (i.e., internalization pathway, intracellular localization, toxicity, etc). Figure 1 reports the representative structure of pristine G (panel a), GO, and G-Red with their typical defects in their basal planes (panels b and c) and FLG (panel d). The functionalization of the surfaces of graphene materials (panel e, Figure 1) with diverse functional molecules such as drugs [5], natural compounds [6], biomolecules [7], polymers [8], metal nanoparticles [9], by covalent and/or not covalent approaches gives rise to graphene family namely Functional Graphene Nanomaterials (FGN). The diverse and exceptional physicochemical and biological properties of FGN originated from the synergistic combination of graphene-based materials related properties, such as high specific surface area of 2D planar nanosheet structure, high availability of surface functional groups, tunable electrical conductivity and mechanical properties, with good biocompatibility and versatile biofunctionality make them a potential platform for multifunctional biological applications. The applications of FGN in nanomedicine have been discussed in numerous reviews [2,10] but all of them are mainly focused on the preparation of FGN and on their biological responses, whereas the activation of cellular signaling pathways by FGN is only marginally or fragmentally reviewed. After a general description of FGN and their main applications in nanomedicine, the review will deeply deal with their cellular uptake mechanisms, the localization into intracellular organelles, and the signalosome activation.

## 2. Functional Graphene Nanomaterials and their Biomedical Applications

In the literature, the term “Graphene” refers to a broad family of graphene products that include, among others, reduced graphene oxide, graphene oxide, graphene nanoplatelets, etc. They possess a different structure and very diverse physicochemical and mechanical properties that affect the biological responses. In addition, these graphene products, in turn, are not homogeneous materials, rather a mixture with various oxidation states, different lateral sizes, varied number of layers, and different colloidal stability. The lack of standardized ways of reporting the characterization of graphene materials and the presence of traces of impurities (often metal impurities are the solely responsible for the toxicity) make difficult, sometimes, the reproduction of published biological experiments [11]. In addition, the combination of graphene products with biomolecules or metal nanoparticles by covalent coupling reactions and/or non-covalent interactions originates new graphene platforms with unprecedented biological properties with respect to the starting materials.

Taking in account the impossibility to give a univocal classification to the broad family of graphene products and the presence in literature of comprehensive recent reviews that describe the different aspects of biomedical applications of graphene [11,12,13], the present review deals with the activation of intracellular molecular pathways induced by the specific functionalization of the graphene platform.

In the last years, the applications of FGN in biomedical fields have prompted great interest and a wide number of compounds have been exploited to convey specific biological properties to graphene materials. Among the different chemical modifications of graphene-based materials, some of them have been designed to generate FGN targeted to mammalian cells (i.e., macrophages, lymphocytes, cancer cells, stem cells etc.), bacteria, yeast, and viruses.

The combination of graphene-based materials with polymers has been tailored to synergistically combine the characteristics of both components to obtain multifunctional nanocomposites with a superior performance for biomedical applications [8]. The functionalization of graphene-based materials with polyethylene glycol (PEG) was proposed to increase the stability in the physiological milieu, to minimize the interactions with proteins and to reduce the risk of immunological response. It is demonstrated that PEG units grafted to GO avoid the formation of intracellular reactive oxygen species (ROS) and trigger mitochondria-related apoptosis [14].

The integration of graphene derivatives with hyaluronic acid (HA)-based polymers has been proposed to obtain intrinsically targeting carriers for specific and controlled delivery of multiple therapeutic agents in the same area of the body and at a predefined extra/intracellular level. The last strategy takes advantage of the selective interactions of HA with specific receptors, such as CD44 (cluster of differentiation 44) and RHAMM (hyaluronan-mediated motility receptor), typically overexpressed on the surface membranes of various tumor cells [15]. The recognition ability of sugar scaffolds and the unique 2D large flexible surface area of the graphene materials were integrated to generate FGN that are able to wrap large-sized microorganism. A good example was reported by Qi et al. that demonstrated the ability of heptamannosylated β-cyclodextrin assembled to adamantyl-functionalized graphene to kill *Escherichia coli (E. coli)*; after the recognition and capturing of *E. coli* by mannose ligands anchored on the platform, the near-infrared (NIR) laser irradiation induced a complete microbial elimination (>99%) [16].

Dendritic polyglycerol sulfate grafted on a graphene surface was proposed as mimicking of sulfated glycol-structures present at the cell membrane. The ability of the obtained FGN to disinfect the orthopox-virus demonstrated that large sheet-like inhibitors could be more effective on the pathogen disinfection, while free dendritic polyglycerol sulfate showed no effect. Probably, the larger contacting area at graphene interfaces blocked the interactions of the entrapped virus with other biological interfaces [17].

The grafting of polymers with different charges at the surface of graphene sheets was used to manipulate the physicochemical properties of graphene (surface charges) and to control the drug intracellular release. Polyglycerol amine and polyglycerol sulfate were employed to give opposite surface charges. The authors reported that both positively and negatively charged graphene derivatives were internalized into lysosomes and then released doxorubicin (Dox) in a different way. The release and efficiency of Dox from the positively charged graphene was much faster than that from negatively charged graphene [18].

Graphene functionalized with a cationic polymer such as polyethylenimine (PEI) has been exploited in gene delivery due to the strong electrostatic interactions of the polymer with negatively charged phosphates of RNA and DNA. Dual polymer functionalized graphene platforms, GO-PEG-PEI and G-Red-PEG-PEI, were synthesized starting from PEGylated GO/G-Red and polyethylenimine (PEI, 25 kDa) and their efficiencies as single or integrated components (i.e., PEI, GO−PEI, GO−PEG−PEI, G-Red-PEG-PEI) as gene delivery systems were compared. Both GO platforms (GO−PEI and GO−PEG−PEI) were endowed of a relatively high transfection efficiency and a low cytotoxicity, but in the absence of the PEG component, the precipitation phenomena in the presence of saline or serum limited the bioapplications [19]. Under NIR irradiation, due to the photothermal effect of GO, the gene delivery efficiency is significantly improved and this effect was found more evident for the reduced platform G-Red-PEG-PEI [20].

The biological behavior of natural compounds grafted on graphene materials was investigated by different research groups and many examples have been reported. The natural flavonoid Silibin (Sil) was linked to G-Red and the activities towards human mesenchymal stem cells (MSCs) and human osteosarcoma cells (MG63) have been compared to free Sil. The inhibitory effects against MG63 were found comparable for both Sil and G-Sil; interestingly, after conjugation, Sil did not affect the viability of MSCs; probably a delayed uptake process, cell type-dependent, could reduce the G-Sil cytotoxicity on MSCs respect to free Sil [21].

The interactions of graphene-based materials with human stem cells have also been deeply investigated in the fields of tissue engineering and regenerative medicine. The ability of graphene platforms to support and, at the same time, to accelerate the growth and proliferation of different types of stem cells was reported by different and independent research groups [22]. From these data emerged important pieces of evidence: (i) graphene acts as a pre-concentration platform of several growth factors and differentiation chemicals, in virtue of its ability to interact with biomolecules (i.e., π–π stacking, hydrogen bonds, electrostatic interactions); (ii) the functional groups on graphene can drive the specific differentiation of different types of stem cells into specific tissue lineages (i.e., fluorinated graphene accelerates the neuronal differentiation of MSCs).

The mechanical and electrical properties of graphene materials can be useful in reinforcing tissue engineering scaffolds; within this application, special attention should be given to the possibility of covalently grafting of peptides, proteins, and growth factors to the surface of the scaffolds, which would act as attractive signals for cells and promote the regeneration process.

The recent literature indicates that graphene-based composites interfaced with micro/nanofabrication technologies may lead to the development of scaffolds with properties fine-tuned for target organ/tissues [23]. However, along with detailed in vitro characterization of scaffolds, more attention should be focused on their evaluation in vivo with respect to inflammatory responses, biocompatibility, and regenerative potential.

New hybrid materials endowed with outstanding properties for theranostic applications were obtained by loading metal nanoparticles (NPs) on graphene platforms [9]. Functionalized iron oxide loaded on graphene was developed as a negative contrast agent for magnetic resonance imaging (MRI), for therapy with magnetically induced hyperthermia and for cell labeling. The properties of gold nanoparticles (AuNPs), such as the strong absorbance from the visible to NIR regions due to the surface plasmon resonance effect, have been exploited for surface-enhanced Raman scattering (SERS)-related applications [24].

Ultrasmall plasmonic gold nanorods and G-Red have been used to develop hybrid vesicles for the remote-controlled release of Dox, loaded at both the cavity of the vesicle and at the surface area of G-Red. The excellent photothermal and photoacoustic properties, due to the G-Red response and the plasmonic effect of gold nanoshell, when irradiated with a NIR laser, guaranteed a dual photo- and acid-responsive Dox release pattern [25].

The ability of graphene-based materials to provide a high capacity of drug loading for many therapeutic agents has been exploited to design multifunctional drug delivery systems for combined therapies: a new trend of the modern nanomedicine (“combo” nanomedicine) [26] has been recently reviewed by Cicchi and co-workers [27].

## 3. Functional Graphene Nanomaterials Cellular Uptake

**Figure 2:**
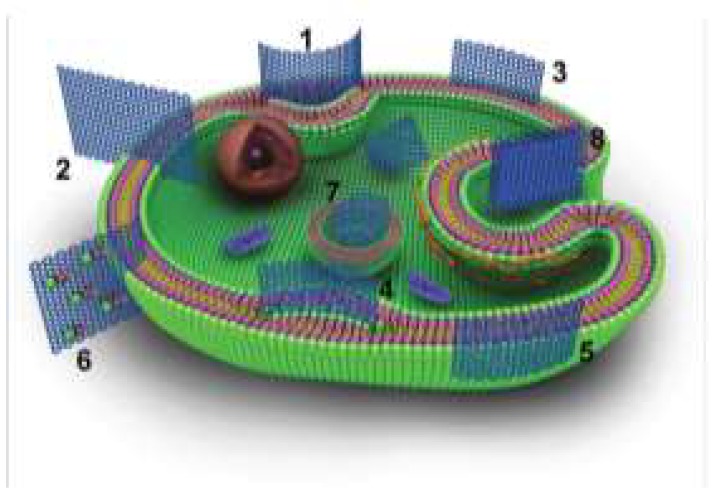
The schematic representation of the possible interaction states between graphene and cell: (1) hemisphere vesicle structure, (2) lying across the membrane (3) graphene-sandwiched superstructure, (4) flat vesiculation, (5) adhering to the membrane surface, (6) destructive extractions of lipid molecules from the membrane, (7) cytoplasmic internalization, and (8) actin filament mediate endocytosis. Partially reproduced from Reference [30], with permission of the Royal Society of Chemistry (RSC).

A fundamental issue for biomedical applications of FGN is the correlation among the physico-chemical properties, the interactions with the plasma membrane, and the cellular uptake mechanism [28]. The interaction of graphene with the plasma membrane is significantly influenced by the size, shape, and surface chemistry (oxidation status, O/C ratio). Hydrophilic GO is manly internalized by endocytosis, while hydrophobic G-Red is mostly adsorbed at the cell surface without internalization [29]. Yan and coworkers [30] recently reviewed the physico-chemical principles of the interaction between graphene and the cell membrane using computational and theoretical approaches, revealing that it strongly depends on the 2D geometry, the surface properties (particularly, oxidation and O/C ratio), and the mechanical stiffness of the sheets. The authors interestingly summarize four essential states of graphene-cell membrane interactions (Figure 2), i.e., graphene-sandwiched (3), hemisphere vesicles (1), lying across the membrane (2), and flat vesiculation (4).

A graphene nanosheet with a lateral size slightly larger than the membrane thickness deforms the membrane, leading to a hemisphere vesicle structure [30]. The GO, with enough oxidization degree and a large side length, can pierce through the membrane and forms the configuration lying across the membrane, with a hydrophobic attraction between the lipids of the membrane and the hydrophobic sections of GO. Pristine or sparsely oxidized graphene nanosheet prompts strong hydrophobic attraction with a small lateral size to insert into the center of the bilayer. When the graphene nanosheet is densely oxidized, its insertion is inhibited, resulting in it being anchored at the membrane surface by adhesion with the top hydrophilic region. A flat-vesicle state with GO wrapped by the membrane occurs in the presence of clathrin, caveolin, and ligand-receptor binding, usually accompanied by vesiculation. All these interaction states present the edge-first uptake where the graphene corners or asperities pierce into the membrane spontaneously, with different insertion speed dependently on the surface properties/oxidation state and the strength of interaction with the hydrophobic/hydrophilic head of the membrane [30]. Regarding the graphene size effects, small hydrophobic graphene nanosheet pierces through the membrane and navigate the double layer; intermediate-size sheets pierce the membrane only if a suitable geometric orientation is met; and larger sheets lie mainly flat on the top of the bilayer where they wreak havoc with the membrane [24]. The thickness (layers number) also influences the graphene-cell interaction: the thinnest nanomaterials (e.g., monolayer G or GO) are quite deformable, while the graphene multilayer may act as a rigid structure during their cellular interactions [30]. Liao and coworkers investigated the effects of graphene size on human erythrocytes revealing that nanosized graphene (350 nm) could induce severe hemolysis attributed to the strong electrostatic interactions with the lipid bilayer of the erythrocyte membrane, compared to microsized graphene sheets (3 μm) which possess relatively low toxicity, likely due to their lower overall surface areas [31].

Endocytosis is the major transport mechanism used by nanosized materials like FGN to cross the plasmatic membrane and it is classified in phagocytosis and pinocytosis [29]. All types of phagocytic cells (monocytes, macrophages, and neutrophils) incorporate large particles through the phagocytosis mechanism, instead, pinocytosis processes involve ingestion of fluid and solutes, according to the involved proteins [32]. Therefore, we can distinguish clathrin-dependent endocytosis, caveolae-dependent endocytosis, macropinocytosis, and clathrin- and caveolae-independent endocytosis [32].

The surface charge of drug-loaded graphene is the main parameters that affect the type of endocytosis pathways and consequently the drug intracellular release and therapeutic efficiency [18].

The cellular uptake of amine-functionalized graphene occurred both through phagocytosis and clathrin-mediated endocytosis, independently of the size, and it resulted in being more efficient compared to negatively charged sheets due to their stronger affinity to the cell membrane. For the negatively charged graphene, it strongly depends on the size of the sheets: large graphene sheets (around 1 μm) underwent mostly phagocytosis, while this pathway was less effective for smaller sheets (200 nm) that are internalized into the cells by clathrin-mediated endocytosis [28].

The functionalization of FGN can influence their interaction with cells not only by affecting the surface properties in terms of hydrophobicity/hydrophilicity but also by promoting a specific interaction with receptors (targeting). The functionalization of G-Red with the surfactant poly (sodium 4-styrenesulfonate) (PSS) was proposed as a tumor-targeted nanocarrier for epirubicin (EPI). The uptake of the platform PSS-G–EPI in human breast cancer cell lines (MCF-7) took place mainly by the endosome-mediated endocytosis pathway, which is slower than the direct diffusion of free EPI into the tumor cells. In fact, after internalization, the nanocarrier is transferred to lysosomes in 2 h, then released in the cytoplasm in 8 h, and eventually delivered into the cell nucleus to exhibit biological effects in 24 h [33]. The functionalization with dextran on G-Red surfaces was proposed to obtain an antigen delivery carrier for cancer immunotherapy with good water dispersibility, high antigen loading, and efficient cellular uptake via endocytosis by interaction with carbohydrate receptors on the surfaces of dendritic cells [34].

Tan and coworkers recently described the cellular uptake mechanism of aptamer-functionalized graphene-isolated-Au-nanocrystals (GIAN) [35]. GIAN is a core–shell structure, in which a thin layer of graphene is grown on the surfaces of gold nanocrystals, designed for combined drug delivery, imaging, and NIR induced hyperthermia. The cellular uptake was investigated under different incubation conditions in HeLa cells, suggesting that the accumulation of GIAN kept increasing with time, reaching the maximum level at 10 h. Different endocytosis pathways were observed: aptamer-functionalized GIAN enter the cells through a clathrin-mediated transport, while GIAN without aptamer modification is internalized by caveolae-mediated endocytosis [35].

A mechanism such as endocytosis induced via cell surface receptor activation emerged as a good strategy to enhance cellular uptake and delivery of therapeutic/imaging agents into specific cells. Sitharaman and coworkers demonstrated that graphene nanoribbons, water dispersed with the amphiphilic polymer PEG-DSPE ((1,2-distearoyl-sn-glycero-3-phosphoethanolamine-*N* [amino (polyethylene glycol)]), activated the epidermal growth factor receptor (EGFR) by inducing a transient membrane depolarization and consequent influx of Ca^2+^ from the extracellular space, leading to an enhanced cellular uptake of the drug-loaded nanoparticles via pinocytosis into tumor cells that over-express EGFR (i.e., HeLa cell lines) [36].

The endosomal escape remains as the “holy grail” for endocytosis-based intracellular drug delivery. To achieve an efficient endosomal escape, a light-responsive liquid-metal “nanotransformer”, constituted by the eutectic alloy of gallium indium (EGaIn) coated with graphene quantum dots (GQDs) has been reported [37]. The nanospheres resulted in being able to absorb photoenergy and generate local heat and ROS that drive the simultaneous phase separation and morphological transformation of the inner liquid-metal core. The morphological transformation from nanospheres to hollow nanorods disrupted the endosomal membrane, promoting endosomal escape and efficient intracellular delivery of payloads [37].

## 4. Subcellular Localization of Functional Graphene Nanomaterials

The intracellular uptake of FGN triggers the activation of intracellular trafficking mediated by endocytic vesicles driving the compartmental flux and the final destination of internalized materials. The endosome/lysosome system is pivotal to the intracellular transportation and entrapment of FGN [32]. The endosome/lysosome system is a set of intracellular extremely dynamic organelles including early endosomes, recycling endosomes, late endosomes, and lysosomes. In addition, autophagosomes execute autophagy, which delivers intracellular contents to the lysosome. Their dynamic interconversion makes the distinction between them often difficult, even with electron microscopy analysis of immunolabeled thin section.

**Figure 3:**
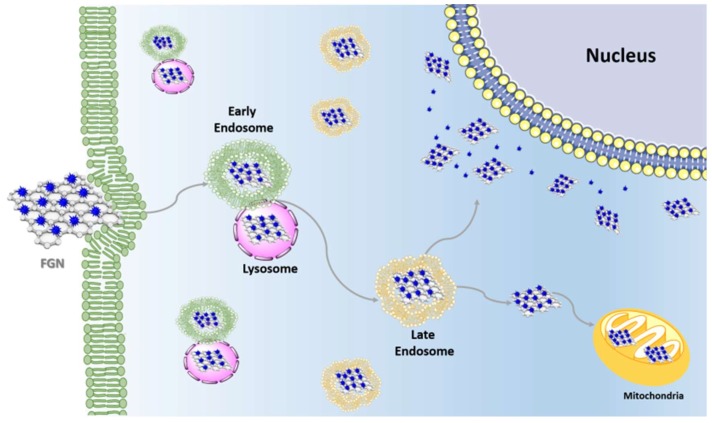
The schematic representation of intracellular transportation and the entrapment of Functional Graphene Nanomaterials (FGN) by endosome/lysosome system.

Different studies have demonstrated that carbon nanomaterial/drug delivery systems were transported by endocytosis and subsequently delivered, intact, by early and late endosomes within the cells (Figure 3) [38,39]. This transport has the advantage that it does not encounter direct drug pumping out from the cytosol and can increase drug retention time through endosome/lysosome delivery [40].

The integrity of lysosome compartment is dependent by FGN concentration; no significant effects on the integrity of the lysosomal membrane have been reported at low FGN concentrations (20 μg/mL), while the membrane permeabilization can be affected at high FGN concentrations (100 μg/mL), which can result in cell death via different pathways, including lysosome/mitochondria-dependent apoptosis, lysosome-dependent necrosis, and lysosome-dependent autophagy) [32]. Autophagy is an essential catabolic process for maintaining cellular homeostasis because it involves a highly dynamic quality control mechanism to degrade aberrant materials and recycle cellular components. The lysosomal localization is tightly correlated to the cellular autophagy processes and several studies have described graphene derivatives as inducers or inhibitors of autophagy [41].

Combined approaches can be used to monitor the activation of autophagic flux and the autophagosome accumulation induced by graphene exposition. That accumulation can be related to an increased autophagosome synthesis or to decreased autophagosome turnover [42].

The microtubule-associated protein 1A/1B-light chain 3 (LC3) is used as a biological marker to identify autophagy induced by graphene exposition. During autophagy, cytosolic LC3-I is converted to LC3-II and associates to the autophagosomal membrane. Thus, the levels of the LC3II protein, the GFP (green fluorescent protein)-LC3 dot number and the treatment with chemical autophagy inhibitors are employed to determine cellular autophagosome accumulation. In addition, to monitor the autophagic flux activation, the p62 expression levels, normally reduced during the autophagy process, can be evaluated. After the graphene internalization by endocytosis, an increase of LC3II on the autophagosomal membrane corresponds to the accumulation of the p62 protein, indicating the autophagy induction mediated by graphene which does not evolve in the degradation of engulfed contents in the autolysosome vesicles. Further studies should be carried out to clarify why graphene or another type of nanomaterials are sequestrated into lysosomal organelles but not degraded. The consequences of graphene on autophagy are not completely clarified and different effects have been reported. Ji et al. demonstrated that the increased accumulation of autophagosome after GO quantum dots treatment resulted by the block of autophagy flux due to the low degradative activity of lysosomes [42]. In another case, the non-canonical activation of autophagy mediated by graphene nanosheets loaded with cisplatin has been reported.

The anticancer drug delivery system induces the initiation of autophagy, which precedes cellular death. In particular, the nuclear translocation of either the LC3 autophagic marker and the chemotherapy drug is stimulated and results in the shut off of the late autophagy events and activation of cell death signals [43].

Deeper studies on the molecular dynamics related to the graphene nanomaterials exposition are still necessary in order to understand whether the graphene endosomal trafficking concludes with lysosomal compartmentalization or involves the recycling of unengulfed materials.

Recently, particular attention has been devoted to the development of drug delivery strategies targeting subcellular organelles such as mitochondria [44]. Anticancer drugs such as Dox, a cytotoxic anthracycline antibiotic that acts mainly into the nucleus by intercalation with the double helix DNA, have also been investigated for their specific actions on mitochondria. It is evidenced the ability of Dox to intercalate mitochondrial DNA perturbing mitochondrial structure and function in tumor cells; this mechanism is also pertinent with the side-effects of Dox in the cardiac tissue. To study the specific activity of Dox on the mitochondria, FGN bearing mitochondria-targeting-ligand such as glycyrrhetinic acid (GA, a pentacyclic triterpenoid isolated from *Glycyrrhiza glabra*) loaded with Dox has been developed (FGN-GA/Dox). From these studies emerged that after internalization, FGN-GA/Dox is transported from the early endosome, gradually maturing into a late endosome and eventually into a lysosome that subsequently transferred mainly to mitochondria due to the targeting properties of FGN-GA. About 82.5% of Dox was delivered into mitochondria and 11.7% of Dox was delivered into the cell nucleus. The FGN-GA/Dox-induced mitochondria-mediated apoptosis (MMA) of cancer cells and exhibited greater antitumor activity compared with Dox·HCl in vitro and in vivo [45]. The nuclear effects of Dox have been maximized using the strategy called “photochemical internalization” (PCI). Due to their strong NIR optical absorption ability, graphene-based materials have been exploited as stimuli responsive-drug delivery platforms activated by the photochemical disruption of the endo/lysosomal membrane. In this direction, a redox-sensitive GO-based nanosheet (HA-SS-GO) was synthesized and used as a Dox carrier. HA-SS-GO/Dox was efficiently internalized into HA-receptor overexpressed tumor cells due to a combination of both passive and active targeting and it was accumulated into endo/lysosomes, where the HA layer underwent digestion by hyaluronidase. Subsequently, NIR irradiation induced the endo/lysosome disruption and the delivery into the cytoplasm, a region with high glutathione (GSH) concentration. Finally, the disulfide bonds of HA-SS-GO were rapidly cleaved through thiol-disulfide exchange allowing for the accelerated release of Dox into the cytoplasm. The accumulation of released Dox into nucleus induced DNA damage-mediated apoptosis and cytotoxicity [45].

The targeted drug delivery strategies have also been investigated to maximize the anticancer effects of photothermal therapy (PTT) or photodynamic therapy (PDT). The basis of these therapeutic strategies is that light is utilized to activate a non-toxic photosensitizer (PS), provoking the formation of heat and highly toxic ROS, mainly singlet oxygen, ^1^O_2_, at the targeted site of cancerous tissue [46]. A PS linked to graphene nanohybrid showed improved anticancer PDT or PTT performance compared to the conventional PS due to the strong surface plasmon absorption upon irradiation [47].

A subcellular targeting for PDT was achieved by the conjugation of GO with integrin αvβ3 monoclonal antibody; after endocytosis, the escape of the nanosystem from lysosomes and the transfer to the mitochondria was observed. The PS (i.e., Pyropheophorbide-a) loaded on the nanosystem became in the “on” state into mitochondria performing its effective phototoxicity to kill cells by increasing the MMA [47].

It was demonstrated that the mitochondrial structure is affected by treatment with GO at low concentrations (<4 μg/mL), resulting in a decline in the membrane potential and the dysregulation of mitochondrial Ca^2+^ homeostasis [48]. Considering that, ROS shows a very short lifetime (less than 4 μs for ^1^O_2_ in aqueous media) and a small diffusion radius (about 0.2 μm for ^1^O_2_) in biological system [49], the spatial control of PS delivery to the primary site of mitochondria in PDT is particularly desirable to induce the efficient local photo-damages. The negative potential gradient of the mitochondrial membrane potential (ΔΨm) (−120 to −170 mV negative inside) addresses nanosystems with positive charges and high lipophilicity to internalize in mitochondria. Furthermore, ΔΨm in most cancer cells resulted in levels higher than that in normal cells. In this framework, a study has demonstrated as a mitochondria-targeted and NIR light-activable multitasking nanographene provoked phototoxicity in situ (by combining PDT and PTT) and immunotherapy. Indeed, GO was functionalized with triphenylphosphonium (TPP) to make a vehicle that was able to address the NIR dye IR820 on the mitochondria, after the lysosomes escape. The high lipophilicity of TPP with three aromatic rings and a positive charge on phosphorous enhanced the cell association and access to mitochondria. Further, the introduction of the immunostimulatory conjugate (i.e., cytosine−guanosine oligodeoxynucleotides) increased the production of proinflammatory cytokines (i.e., interleukin-6, tumor necrosis factor-α, and interferon-γ), thus, modulating the tumor immunogenicity [50]. From experimental data emerged that target nanophototherapeutics, generating ROS and heat, ultimately kill cancer cells by inducing mitochondrial collapse and irreversible cell apoptosis upon NIR laser irradiation and the target systems prompted much higher early apoptosis than non-target counterparts. 

Recently, in order to optimize the PDT effectiveness and therapeutic efficacy by increasing ROS production directly in mitochondria, a rare-earth doped upconversion nanoparticle (UCNP) was coupled with GQD and a rhodamine derivative (i.e., TRITC, tetramethylrhodamine-isothiocyanate) for mitochondrial targeting. The system exploited the properties of UCNP, which can emit UV/VIS light under NIR excitation, and GQD, which can efficiently generate ^1^O_2_. Hence, mitochondrial-specific PDT with in-situ ^1^O_2_ burst leads to the decrease of the mitochondrial membrane potential inducing irreversibly tumor cell apoptosis [51].

Although the mechanism is not clear, the nuclear sub-localization of graphene-based materials has been ascertained in some cases [52,53]. The comprehension of the cytoplasm-nucleus shuttle effect of graphene compounds might be useful to design carriers for drug and gene delivery.

The analyses of the literature data suggest that it is hard to predict the exact internalization mechanisms and the subcellular localization of graphene derivatives. Due to having a great diversity of structure, dimension, charge and colloidal stability, the cellular uptake is probably the result of the combination of different endocytotic pathways that might be associated with passive internalization.

The studies on the different internalization pathways and the intracellular fate of graphene nanocarriers should be in deep, since they may enlighten the research on organelle-targeting drug delivery systems.

## 5. Intracellular Molecular Pathways Activated by Functional Graphene Nanomaterials

The intracellular internalization of graphene-based materials triggers a wide variety of cellular responses mediated by the activation of the intracellular signaling network, which affects the cell environmental [54,55,56]. The expression of cytokines, growth factors, and gene regulatory proteins emerges from the ability of nanomaterials to modulate a cellular pathway such as apoptosis, cell cycle, inflammation, and metabolic processes [57]. Therefore, nanomaterials delivery can potentially interfere with diseases or disorders closely related to defects in specific intracellular pathways [58,59]. Graphene and its derivatives represent an increasingly attractive delivery system and the strategy of covalent and non-covalent functionalization seems to increase the stability, the solubility, the biocompatibility and biological applications [60]. On the one hand, graphene decoration influences the cell growth, the ROS release or the secretion of nitric oxide (NO) in order to differentiate cancer cells and normal cells [33,61,62,63,64,65,66]. Graphene functionalization can promote drug delivery and increase the tissue specificity of the delivery systems in order to enhance the drug bioavailability and overcome multidrug resistance in conventional cancer therapy [67,68,69]. The GSH levels reduction, the lipid peroxidation, the alteration of antioxidative enzyme gene expressions (SOD1, SOD2, CAT, GSTA1, and GSTA4) [29] and mitochondrial depolarization [45] are induced by graphene-mediated overproduction of ROS. Several groups have demonstrated that the ROS-induced high toxicity depends on the size and surface functionalization of graphene-based materials, demonstrating that the reduction of oxygen functional groups on the graphene surface minimizes the cytotoxicity and increases the biocompatibility [70]. The ROS and NO production results on the downstream activation of the transcriptional factor NF-kb, proinflammatory cytokines (TNF-α, IL-1β, or IFN-γ) and cellular kinases involved in the promotion of cell proliferation. Studies in vivo have demonstrated that the G-Red-dextran enhanced the immunostimulatory capability of dendritic cells (DCs) triggering the inflammatory cytokines release and the subsequent activation of cytotoxic T cells [34].

The modulation of immune response related to graphene exposition was studied in primary murine and immortalized macrophages. Zhou and collaborators [71] have demonstrated that lower concentrations of pristine G increased the transcriptional levels and the expression of cytokines and chemokines which activated proinflammatory signals and shut off their expression to avoid the effects of an overactivation related to graphene exposition. The proinflammatory stimuli were activated by the upstream interaction between the Toll-like receptor and graphene sheets, which stimulated the NF-κB nuclear translocation and promoted the transcription of the proinflammatory gene. The proinflammatory cytokines release influenced the neighboring macrophages morphology by inducing an F-actin rearrangement resulting in a limited macrophages adhesion to the inflammatory site. In addition, the clearance of infections and the removal of cellular debris is macrophage-mediated. The graphene-exposition limited the phagocytosis ability and opened a new prospect on the use of graphene on the regulation of inflammatory disease and tissue lesions due to the unbalanced production of inflammation mediators [71].

A potential branch of cancer gene therapy is represented by immunotherapy approach that stimulates directly the immune response against cancer. Therefore, the evaluation of the impact of the graphene sheets on the cellular environment and the potential modulation of the key components of the immune system could be interesting for a new immunotherapy approach. It has been found that the lateral dimensions of GO can modulate the expression of immune genes encoding for chemokines, cytokines and cytokine receptors. A deep and interesting study by using a genomic approach was conducted by Orecchioni M. et al. [72] on human peripheral blood mononuclear cells (PBMCs). They analyzed the differential expression of immune genes following the exposition to large and small GO sheets. Immune gene expression array showed the upregulation of genes related to inflammatory response and encoding for IL1R1, IFNAR1, CSF2, TNF, CCL5, IL6, IL1α, IL1β, and IL8 following small GO sheets treatment, compared to large GO sheets. These finding suggested that the GO structure affects the capability to internalize into the cells, influencing the immune gene expression profiling and triggers different biological effects [72]. Recently, the FLG treatment showed a specific toxicity on monocytic neoplastic cells. Preliminary results demonstrated in vitro that the monocytes population decreased drastically at an early time point after FLG exposition involving the necrosis processes not stimulated by the activation of T cells. The whole-genome gene expression profiles revealed the upregulation of genes encoding for several chemokines and cytokines following FLG exposition. The administration of FLG on primary human monocytes reduced drastically the neoplastic cell number highlighting the capability of graphene to target and kill monocytoid cancer cells [73].

Single-cell mass cytometry analysis in combination with whole-transcriptomic analysis was performed on purified PBMCs treated with GO and amino GO. The amino functionalization on GO reduced the necrotic events, incremented the CD25 and CD69 expression levels in active T cells, as well as the gene expression for pro-inflammatory cytokines, regulators for an innate and adaptive response. These data suggested the enhanced biocompatibility of GO due to functionalization [74].

Current knowledge has demonstrated that the graphene nanostructure triggers an in vitro programmed cell death mechanism in a dose-dependent manner. During earlier events of apoptosis, the translocation of phosphatidylserine occurs from inner to the outer leaflet of the plasmatic membrane. The fluorochrome-labeled Annexin V analysis allows detecting the interaction between Annexin V and the phosphatidylserine exposed on the outer surface, through a flow cytometry assay. Tabish et al. showed that lung cancer cells exposed to a lower concentration of G-Red exhibited late stage of apoptosis, rather than early stages, with a consequent formation of the apoptotic bodies and damage of cell membrane [74]. Instead, Kang et al. performed a comparative analysis between intracellular molecular signaling activated by the exposition of GO and G-Red on neuronal cell lines (PC12), showing significant levels of apoptosis induced by higher doses of GO rather than G-Red [75]. Probably, the differences related to chemical propriety impacted on the molecular mechanism activated by the graphene exposition. In addition, extrinsic or intrinsic death signals stimulated the release and the activation of the mediators of apoptosis. In particular, the exposition to graphene seemed to increase the mitochondrial membrane permeability, provoking the release of apoptosis-inducing factor. Li et al. have demonstrated that the intrinsic apoptosis pathway (mitochondrial-mediated) represented the main death mechanism triggered by pristine G [76]. In particular, the mitochondrial outer membrane depolarization after graphene exposition occurred in a time-dependent manner and resulted in the increment of expression levels of apoptotic effector enzyme, such as caspase-3. Pristine G treatment promoted the accumulation of a cleaved form of nuclear protein PARP (poly-ADP-ribose polymerase), a substrate for caspases and a biomarker for the activation of cellular death programs. The accumulation of pro-apoptotic proteins, Bax and Bim, the consequent mitochondrial permeabilization, the release of cytochome c from the mitochondrial to cytoplasmic side, and the downstream activation of caspase 3 occurred following pristine G induction and according to a mitogen-activated protein kinase (MAPK)-dependent mechanism [77]. The involvement of the MAPK cascade also occurred in the context of the cell cycle and among the MAPKs, the ERK (extracellular signal-regulated kinases) protein interacted with a network of downstream kinases and regulated the cellular outcome. Neuronal ERK signaling activation was detected in response to GO in the nematode *Caenorhabditis elegans*. In addition, Kang et al. [76] have demonstrated that GO and G-Red seemed to be able to arrest cell cycle progression in tumor neuronal cell lines and shaped the ability of the cell to normally divide by involving the decreasing MEK1/2 and ERK phosphorylation state following GO and G-Red treatment. These findings highlight the importance of functionalization in the enrichment of the biocompatibility of graphene-based nanomaterials.

## 6. Conclusions

Graphene-based materials have emerged as a subject of enormous scientific interest because of its exceptional properties and they have been proposed for various biomedical applications, such as biosensors, tissue engineering, and drug delivery. However, until now, to the best of our knowledge, no graphene-based materials have progressed to the clinical stage. The main obstacles to this advancement are the non-homogeneous nature of graphene materials and the lack of standardized ways of reporting their characterization. Moreover, along with detailed in vitro characterization of graphene derivatives, more attention should be focused on their evaluation in vivo with respect to inflammatory responses, biocompatibility, and signaling pathways. Thus, the road of graphene in biomedical/pharmaceutical applications seems still very long and winding. However, the enthusiasm of the scientific community is providing a host of very interesting breakthroughs, which place graphene in a pole position in the fields of theranostics and tissue engineering. The present review deals with modified graphene materials in which the structural, physical, chemical, and electronic properties and the biological response (i.e., internalization pathway, intracellular localization, toxicity etc.) are tuned by organic functionalization and highlights the advances in the investigations of FGN in nanomedicine. The interactions of FGN with cells, the different pathways of cellular uptake and the sub-cellular localization have been dealt. The current knowledge about the intracellular pathway activated by FGN appears still fragmentary and the correlations between the graphene surface functionalization and intracellular signaling network are not yet sufficiently clarified. These aspects should be deeply investigated considering the importance of graphene-based nanomaterials to modulate a cellular microenvironment. 

## Abbreviations

AuNPs	Gold nanoparticles
DCs	Dendritic cells
DNA	Deoxyribonucleic acid
Dox	Doxorubicin
EGaIn	Eutectic alloy of gallium indium
EGFR	Epidermal growth factor receptor
EPI	Epirubicin
ERK	Extracellular signal–regulated kinases
FGN	Functional Graphene Nanomaterials
FLG	Few-layer graphene
G	Graphene pristine
GA	Glycyrrhetinic acid
GIAN	Graphene-isolated-Au-nanocrystals
GO	Graphene oxide
GQDs	Graphene quantum dots
G-Red	Reduced graphene oxide
GSH	Glutathione
HA	Hyaluronic acid
MAPK	Mitogen-activated protein kinase
MMA	Mitochondria-mediated apoptosis
MRI	Magnetic resonance imaging
MSCs	Mesenchymal stem cells
NIR	Near infrared
NO	Nitric oxide
NPs	Nanoparticles
PARP	Poly ADP ribose polymerase
PCI	Photochemical internalization
RNA	Ribonucleic acid
PDT	Photodynamic therapy
PEG	Polyethylene glycol
PEG-DSPE	(1,2-Distearoyl-sn-glycero-3-phosphoethanolamine-*N* [amino (polyethylene glycol)]
PEI	Polyethyleneimine
PS	Photosensitizer
PSS	Poly(sodium 4-styrenesulfonate)
PTT	Photothermal therapy
ROS	Reactive oxygen species
SERS	Surface-enhanced Raman scattering
Sil	Silibin
TPP	Triphenylphosphonium
TRITC	Tetramethylrhodamine-isothiocyanate
UCNP	Upconversion nanoparticles
